# Beware of Rhabdomyolysis After a Renal Graft

**DOI:** 10.7759/cureus.30546

**Published:** 2022-10-21

**Authors:** Ghita El Bardai, Basmat Amal Chouhani, Widad Haddane, Nadia Kabbali, Tarik Sqalli Houssaini

**Affiliations:** 1 Nephrology, Dialysis, and Transplantation, Hassan II University Hospital, Fez, MAR; 2 Laboratory of Epidemiology and Health Science Research (ERESS), Faculty of Medicine, Sidi Mohammed Ben Abdellah University, Fez, MAR

**Keywords:** secondary effect, toxicity, renal allograft, tacrolimus, rhabdomyolysis

## Abstract

In recent years, there has been a significant advancement in the field of immunosuppressive therapy in renal transplantation. However, these treatments have side effects, including rhabdomyolysis. In this article, we report the case of a transplant patient with rhabdomyolysis secondary to tacrolimus and shed light on different aggravating factors. Treatment withdrawal, hydration, and forced diuresis are allowed in the majority of cases.

## Introduction

Over the years, the use of immunosuppressants has improved graft survival rates in solid organ transplantation [1]. Nevertheless, these medications are not without the risk of side effects. Rhabdomyolysis is the necrosis of skeletal muscle fibers with an efflux of electrolytes and intracellular proteins into the general circulation. It is life-threatening because it can cause hyperkalemia and acute kidney injury (AKI). Although the etiologies of rhabdomyolysis are numerous, severe forms are most often encountered during muscle compressions [2]. In this article, we report a case of rhabdomyolysis after a kidney transplant following the administration of tacrolimus associated with the administration of mycophenolate mofetil, valaciclovir, and trimethoprim/sulfamethoxazole (TMP/SMX).

## Case presentation

A 25-year-old man, who had been on hemodialysis for 15 years due to tubulointerstitial nephritis secondary to a urinary tract malformation, received a kidney transplant from a 34-year-old cadaveric donor. The donor and recipient were of identical O+ blood type with incompatible human leukocyte antigen (HLA) and anti-HLA-positive antibodies. The crossmatch test and the donor-specific antibody (DSA) serums were negative.

The pre-transplant workup did not reveal any abnormalities. Thymoglobulin was used as an induction agent until the ninth day. The patient also received mycophenolate mofetil (1 g pre-surgery and then 2 g per day post-surgery) and steroids. The resumption of diuresis was immediate. The patient was on 6 mg of tacrolimus per day. The associated treatment was based on valaciclovir and TMP/SMX. The evolution was marked by the increase in creatinine phosphokinase (CPK) levels (10 times the normal range) and lactate dehydrogenase (LDH) levels (358 U/L), without clinical symptoms. No worsening in creatinine levels was noted. The tacrolimus residual rate was high (21.4 ng/L).

**Figure 1:**
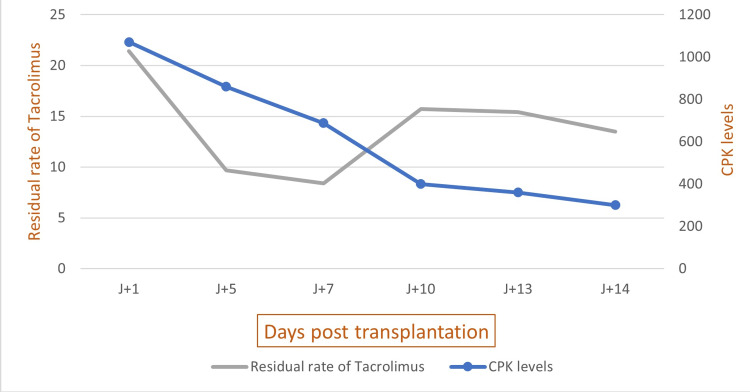
Kinetics of the evolution of CPK values and residual rate after transplantation. CPK: creatinine phosphokinase

The method of French imputability causality assessment was used [3]. The most incriminated drug was tacrolimus, which had an imputability score of C2 for chronology and S2 for semiology, indicating that an intrinsic imputability score of I3 was likely. The extrinsic imputability score was B3. However, mycophenolate mofetil, valaciclovir, and TMP/SMX showed a lower score of imputability. The evolution was marked by an improvement. CPK levels decreased two days after the tacrolimus dose was reduced and returned to normal on the 10th day (Figure 1).

## Discussion

Rhabdomyolysis is characterized by severe muscle destruction and myoglobinuria. Damaged cells release their content into the bloodstream, which leads to a rise in serum myoglobin levels, followed by an increase in CPK levels. The myoglobin is largely eliminated by the kidneys and has a hepatic metabolism that results in a rapid decrease in its concentration and a return to normal levels within one to six hours. The diagnosis of rhabdomyolysis in the early stages can be challenging as myoglobinuria can appear hours before an increase in serum CPK levels [2,4]. Muscle destruction also results in electrolyte release into the blood circulation, leading to hyperkalemia, hyperuricemia, hyperphosphatemia, and early hypocalcemia. AKI is one of the most serious complications of rhabdomyolysis, which is observed in 5-25% of all cases. AKI occurs in up to 16.5% of patients with myoglobinuria [4,5].

In theory, any form of muscle damage can initiate the rhabdomyolysis process. It has been linked to various drugs and clinical outcomes. Drugs and alcohol appear to be the most common causes of rhabdomyolysis in adults (up to 81%) [4]. Other causes include muscle compression in crash syndrome, durable immobility, excessive exercising, alcohol and drug (such as heroin, cocaine, and amphetamines) consumption, hereditary muscle enzyme defects, infectious diseases, diabetic ketoacidosis, and hypo/hyperthermia [6]. Rhabdomyolysis symptoms include muscle injury and/or pain, weakness, tea-colored urine, and kidney injury. However, many patients do not show any symptoms, as was the case of our patient. Our patient’s laboratory examinations showed elevated serum CPK and myoglobin levels. The urine dipstick is positive for myoglobinuria. However, even if it is negative, rhabdomyolysis should not be ruled out as it may be absent in about 50% of the cases. Myoglobin was not found in our patient’s urine [7].

Rhabdomyolysis is secondary to anti-calcineurin inhibitors, as first described in 2015 in a pediatric patient with a bone marrow transplant. His laboratory examinations showed high CPK levels following a period of elevated blood tacrolimus levels, which may indicate that tacrolimus affected skeletal muscle as well [8]. According to Demirbaş et al., rhabdomyolysis accounts for 0.8% of all side effects secondary to calcineurin inhibitors [9]. In this study, there was a temporal relationship between the onset of rhabdomyolysis and the high residual level of tacrolimus. High CPK levels may be attributed to more than one cause. In our patient, TMP/SMX and valaciclovir administration could have contributed to rhabdomyolysis [10,11]. Thus, rapid identification of the etiology of rhabdomyolysis is crucial because any suspected drug must be identified to modify or discontinue treatment.

Recent studies on the polymorphism of genes responsible for the biotransformation enzymes and drug transporters have highlighted new perspectives regarding the individualization of dosages. Genetic factors have been qualified to play an important role in the interethnic and interindividual variability of calcineurin inhibitors [12]. Tacrolimus is generally known to be metabolized by cytochrome P450 3A5 CYP3A5 and CYP3A4 enzymes. Therefore, the pharmacokinetic variability of tacrolimus could be secondary to an allelic variation of *CYP3A5 *and *CYP3A4 *genes [13].

Current treatment of rhabdomyolysis is based on the withdrawal of the toxic agents, proper rehydration by intravenous volume expansion, urinary alkalinization, and forced diuresis. If necessary, hemodialysis or even plasma exchange should be considered [14]. The prognosis of rhabdomyolysis is generally excellent, but it depends on its severity and comorbid conditions. AKI plays an important role as a mortality rate of 52% has been noted in patients with AKI compared with 14% in those without AKI [4].

## Conclusions

The risk of rhabdomyolysis secondary to calcineurin inhibitors increases among patients on concomitant drugs. It is also influenced by other factors such as liver and renal dysfunction. Fast identification and diagnosis based on clinical and biological findings with adequate therapy is crucial and allows recovery and restoration of renal function to baseline. New genetic findings regarding transporter polymorphisms affecting calcineurin inhibitors metabolism should enable individualized medical solutions.
